# Preventing contrast-induced nephropathy: problems, challenges and future directions

**DOI:** 10.1186/1741-7015-7-24

**Published:** 2009-05-13

**Authors:** Richard Solomon

**Affiliations:** 1 University of Vermont College of Medicine, Burlington, VT 05405-0068, USA

## Abstract

Contrast-induced nephropathy is an injury to the kidney occurring as a result of exposure to intravascular contrast media. It results in both short- and long-term adverse events including mortality. Since treatment of the injury after it has occurred is ineffective, efforts to prevent the injury are the focus of investigators and clinicians alike. In this commentary, the pathogenesis and clinical relevance of contrast-induced nephropathy are reviewed. Prophylactic strategies are discussed with a focus on the use of meta-analysis of small single-center trials.

## Introduction

Contrast-induced nephropathy (CIN) is an acute kidney injury associated with both short- and long-term adverse outcomes, including the need for renal replacement therapy, increased length of hospital stay, major cardiac adverse events, and mortality [1-3]. Since there is no effective therapy once injury has occurred, prevention is the cornerstone for all patients at risk for acute kidney injury. There is a small but growing body of evidence that prevention of the acute kidney injury is associated with a reduction in those later adverse outcomes. The article by Meier and colleagues, published this month in *BMC Medicine*, examines the prophylactic treatment of CIN using intravenous bicarbonate for prevention of CIN and the impact of such therapy on long term adverse events[4].

CIN involves at least two complementary pathophysiologic processes. First, contrast media is directly toxic to renal tubule cells, leading to mitochondrial dysfunction, generation of reactive oxygen species (ROS), and programmed cell death [5,6]. Second, contrast media reduces medullary blood flow further compromising a very tenuous balance between oxygen consumption and tissue oxygen availability in this critical area of the kidney. It is this part of the kidney that contains the last part of the proximal tubule and the thick ascending limb of Henle where a large proportion of sodium reabsorption occurs through active transport[7].

## Current limitations of diagnosis of CIN

Our ability to diagnose CIN is limited by the lack of an appropriately sensitive and specific marker of kidney injury. Neutrophil gelatinase associated lipocalin, kidney injury molecule-1, interleukin-18 and other markers are currently being evaluated as reliable indicators of injury and predictors of adverse outcomes [8]. Until such validation occurs, changes in kidney function, specifically glomerular filtration rate (GFR), remain our only means of diagnosing CIN. In clinical practice, changes in GFR are estimated by absolute or relative changes in serum creatinine, that is, increases of ≥0.5 mg/dl or ≥25%, respectively, occurring 48 to 72 hours after contrast exposure. Unfortunately, changes in serum creatinine can occur for reasons other than a decrease in GFR and thus are not specific for a decrease in GFR. Furthermore, because of the time lag between a fall in GFR and a rise in creatinine, the timing of creatinine measurement following contrast exposure can affect the sensitivity of the test [9]. Cystatin C has been advocated as a more sensitive marker of a fall in GFR [10-12].

## Current status of prevention therapies

A variety of failed approaches has led to skepticism regarding our ability to effectively prevent the injury causing CIN. Systemically administered vasodilators, such as dopamine agonists, adenosine antagonists, prostaglandins, and endothelin antagonists, have been disappointing despite the rationale behind their use [13]. Antioxidants, such as N-acetylcysteine, ascorbic acid, and bicarbonate, have enjoyed initial enthusiasm based upon single-center trials [14-16]. However, when considering data presented at society meetings and the increasing number of published negative trials, enthusiasm has waned and even meta-analyses have not found significant efficacy [17]. It is in this context that the article by Meier *et al*., published this month in *BMC Medicine *[4], is particularly valuable as it explores reasons for the heterogeneity in trial results.

## Bicarbonate therapy for prevention of CIN

Bicarbonate therapy was initially explored because the generation of ROS is pH dependent through the Haber-Weiss reaction [18]. It was argued that systemic alkalinization might reduce ROS generation and minimize the subsequent kidney injury. Subsequent studies with acetazolamide, which alkalinizes the urine while causing systemic acidification, showed that it is the urinary space that is the important site at which this pH effect acts [19]. Alkalinization of the urinary space is achieved very quickly with infusion of sodium bicarbonate because normally there is little bicarbonate in the urine. Even a small increase in serum bicarbonate of 1 to 2 mEq/liter will result in the 'dumping' of bicarbonate into the urine in most patients. Such a change in serum is easily obtained with the infusion rates recommended in most of the CIN prevention trials [14,20].

Bicarbonate therapy is readily available, inexpensive, and safe. The question is whether it is efficacious for prevention of CIN. Some single-center trials have found bicarbonate therapy to be efficacious while others have not. A previous metaanalysis of a small number of trials found bicarbonate to be beneficial only when not combined with other prophylaxis [21]. This heterogeneity in results leaves the physician confused at best and apathetic to prevention strategies at worst. Differences in selection criteria of patients, definition of outcome (CIN), protocols for administration of therapy, use of concomitant therapies, timing of follow-up serum samples, and so on may account for this heterogeneity. The article by Meier *et al. *tries to unravel some of these issues by looking at specific subsets of patients. For example, evidence of publication bias was found which the authors tried to overcome by including unpublished trials presented at major clinical meetings. Of particular clinical interest was the finding that bicarbonate therapy was most effective in patients who experienced urgent or emergency contrast exposure. Presumably this selects a group of patients who are less likely to receive any other form of prophylaxis for CIN. This is of great potential importance for the emergency room and cardiac catheterization laboratory. Bicarbonate therapy was also most effective in those receiving low osmolality contrast media compared with iso-osmolality contrast media. Low osmolality contrast is increasingly chosen because of its safety, lower costs, and higher iodine content [22].

## Future directions

CIN needs to be redefined using markers of kidney injury that are sensitive, specific, and predictive of adverse outcomes. This will enable researchers to better address the question of how to prevent and/or treat this condition in the future. The most important question to be answered is whether prevention of kidney injury results in a change in short- and long-term adverse outcomes. Some prevention strategies have been associated with a reduction in long-term adverse events [23,24], while others have not [25-27]. The meta-analyses by Meier *et al. *found that despite a reduction in the incidence of CIN, bicarbonate therapy had no benefit on the need for dialysis or mortality. No matter how available, inexpensive, and safe a therapy, to find an important role in clinical therapeutics, it must improve the 'downstream' adverse outcomes, an as yet elusive goal for the prevention and treatment of CIN.

## Abbreviations

CIN: contrast-induced nephropathy; GFR: glomerular filtration rate; ROS: reactive oxygen species

## Competing interests

RS is a consultant to Bracco Diagnostics and Covidien, pharmaceutical companies which manufacture and sell contrast media.

## Pre-publication history

The pre-publication history for this paper can be accessed here:


